# Prenatal Diagnosis of Clubfoot: Where Are We Now? Systematic Review and Meta-Analysis

**DOI:** 10.3390/diagnostics11122235

**Published:** 2021-11-29

**Authors:** Laura Ruzzini, Sergio De Salvatore, Umile Giuseppe Longo, Martina Marino, Alessandra Greco, Ilaria Piergentili, Pier Francesco Costici, Vincenzo Denaro

**Affiliations:** 1 Department of Orthopedics, Children’s Hospital Bambino Gesù, Palidoro, 00165 Rome, Italy; laura.ruzzini@opbg.net (L.R.); pierfrancesco.costici@opbg.net (P.F.C.); 2 Department of Orthopaedic and Trauma Surgery, Campus Bio-Medico University, Via Alvaro del Portillo, 200, Trigoria, 00128 Rome, Italy; s.desalvatore@unicampus.it (S.D.S.); martilibia@gmail.com (M.M.); alessandra.greco5@gmail.com (A.G.); i.piergentili@unicampus.it (I.P.); denaro@unicampus.it (V.D.)

**Keywords:** congenital talipes equinovarus, CTEV, clubfoot, ultrasound, US, magnetic resonance imaging, MRI, karyotyping, amniocentesis

## Abstract

The primary methods for prenatal diagnosis of Clubfoot are ultrasound (US) and magnetic resonance imaging (MRI). An ultrasound is performed between the 1st trimester and the 28th week of pregnancy and it is reported to be used as a diagnostic method alone or in combination with MRI. So far, an international consensus on the most effective screening method has not been reached. This systematic review and meta-analysis were performed to establish the most effective and reliable exam for prenatal diagnosis of Clubfoot. The literature search was conducted using a PIOS-approach from May 2021 to June 2021. Studies reporting cases of prenatal diagnosis of Clubfoot made through US and MRI conducted from January 2010 to June 2021 were included in the study and reviewed by 2 authors. The 23 selected studies included 2318 patients. A total of 11 of the studies included details on the accuracy, while the rest were used to obtain information about the primary methodology utilized. In all the selected studies, US was used as the primary diagnostic instrument. Thirteen of the studies used the US exclusively, while three used MRI in addition to US and seven performed karyotyping after US diagnosis. The US has been shown to be the instrument of choice for the prenatal diagnosis of Clubfoot. International guidelines for an ultrasonography classification of congenital clubfoot are required to reduce the inter-variability accuracy of this procedure.

## 1. Introduction

Congenital talipes equinovarus (CTEV), or Clubfoot, is a congenital birth defect with a reported incidence of 1–2.5 cases per 1000 births [1]. It consists of a structural abnormality causing an inversion of the forefoot and heel and may be associated with a variety of other birth defects, chromosomal abnormalities, and musculoskeletal disorders [2].

Incidence of Clubfoot varies across geographical regions: Africa, the Americas, and the Eastern Mediterranean have values of 1.1, 1.7, and 2 (cases/1000 birth), respectively [3]. 

The primary methods for the prenatal diagnosis of clubfoot are ultrasound (US) screening, and magnetic resonance imaging (MRI). US detection of Clubfoot has drastically improved over the past two decades; however, there is little literature on specific diagnostic techniques and classification, and false-positive diagnoses remain high with values ranging from 10–40% [4,5]. 

Compared to the US, MRI is not adopted as a screening method due to unclear effects on the developing fetus [6] and the high cost of the procedure. Some authors reported that in the case of positive US, MRI does not advance findings. Therefore, MRI seems to be a suitable method to confirm uncertain US diagnoses [2]. 

In addition to prenatal imaging, which provides a means of screening and detection for the condition, karyotyping may be considered for a complementary investigation to provide a complete diagnosis. This technique detects associated abnormalities [4] and the specimens could be obtained by chorionic villus sampling (CVS) or amniocentesis.

Despite the fact that the treatment of clubfoot and its prenatal diagnosis have been thoroughly investigated by several authors, few high-quality studies focused on the most accurate screening method. Moreover, no international consensus on which examinations should be performed was reported in the literature. Therefore, this review aims to collect available literature on the prenatal diagnosis of clubfoot to determine which screening method is most effective.

## 2. Materials and Methods

### 2.1. Study Selection 

The research question was formulated using a PIOS-approach: Patient (P); Intervention (I); Outcome (O) and Study Design (S). The aim of this study was to establish the most reliable instrument of prenatal diagnosis of clubfoot (P). The latest literature in which prenatal diagnoses of clubfoot (P) was performed using US and/or MRI (I) was reviewed. The diagnostic instrument was then evaluated based on the accuracy (O). The following study designs were included (S): Randomized Controlled Trials (RCT) Prospective (PS), Retrospective (RS), Case series (CS), Case-Control (CC), and Cohort (C) studies. 

### 2.2. Inclusion Criteria

Only articles published in English were considered. Peer-reviewed articles of each level of evidence according to the Oxford classification were screened. Only studies that reported cases of prenatal diagnosis of clubfoot made through US and/or MRI and those that performed karyotyping were included. 

### 2.3. Exclusion Criteria

Technical notes, letters to editors, instructional courses or studies focusing on prenatal diagnosis of pathologies other than Clubfoot were excluded, as well as studies regarding the postnatal diagnosis of clubfoot. Studies dating back to longer than 2010 were not considered to include only the most up to date literature. Studies in which the sample size was smaller than 10 patients were considered ineligible for the present study. 

### 2.4. Search

The Preferred Reporting Items for Systematic Reviews and Meta-analyses (PRISMA) guidelines were used to conduct a systematic review. Medline, Embase, Cinahl, Scopus, Web of Science and Google Scholar were searched as bibliographic databases. The string-searching used was the following: (((clubfoot) OR (talipes)) AND ((((chorionic villus sampling) OR (amniocentesis)) OR (ultrasound)) OR (prenatal diagnosis)) AND ((english[Filter]) AND (2010:2021[pdat]))) NOT (ponseti AND (english[Filter])). Keywords were used both isolated and combined. Two of the authors (G.A and M.M) performed the search from May to June 2021 and articles from January 2010 to June 2021 were screened.

### 2.5. Data Collection Process

The data collection process was performed by two of the authors (A.G. and M.M.) independently. Any disagreement was solved by the consultation of a third reviewer (S.D.S). The screening approach used was the following: A.G. and M.M. proceeded firstly with the review of title and abstract and then of the full-text version. The papers not excluded during the title and abstract screening were evaluated in full text. S.D.S intervened in case of disagreement. The PRISMA flowchart was used to report the number of articles included or excluded. 

### 2.6. Data Items

General study characteristics which were extracted included: primary author, year of publication, country, type of study, level of evidence, sample size, sex, diagnostic instrument, age of gestation at the time of diagnosis, associated pathologies (Table 1 and Table 2).

The accuracy, also known as the percentage of individuals whose prenatal diagnosis of clubfoot was confirmed after birth, was assessed for the studies in which it was specified.

### 2.7. Risk of Bias

The Risk of Bias in Non-Randomized Studies of Interventions (ROBINS-I) and Risk of Bias for Randomized Trials (RoB-2) tools by Cochrane are used to assess the possibility of bias in included studies. The selected articles were independently scored by authors M.M. and A.G. Any disagreements were resolved by a third reviewer S.D.S. 

### 2.8. Statistical Analysis

Categorical data were summarized as frequencies with percentages. Since the high heterogeneity, the subgroup meta-analysis was performed using a random-effect model, and the estimation of the between-study variance was conducted with the Der-Simonian and Laird method. The I^2^ statistic was used to quantify the heterogeneity among the studies, with 50% defined as the threshold for high heterogeneity [28]. A *p*-value less than 0.05 was considered statistically significant. All of the statistical analyses were performed using R software version i368 3.6.1 (R Core Team (2020). R: A language and environment for statistical computing. R Foundation for Statistical Computing, Vienna, Austria. URL https://www.R-project.org/, accessed on 15 November 2021).

## 3. Results

### 3.1. Study Selection

**Figure 1:**
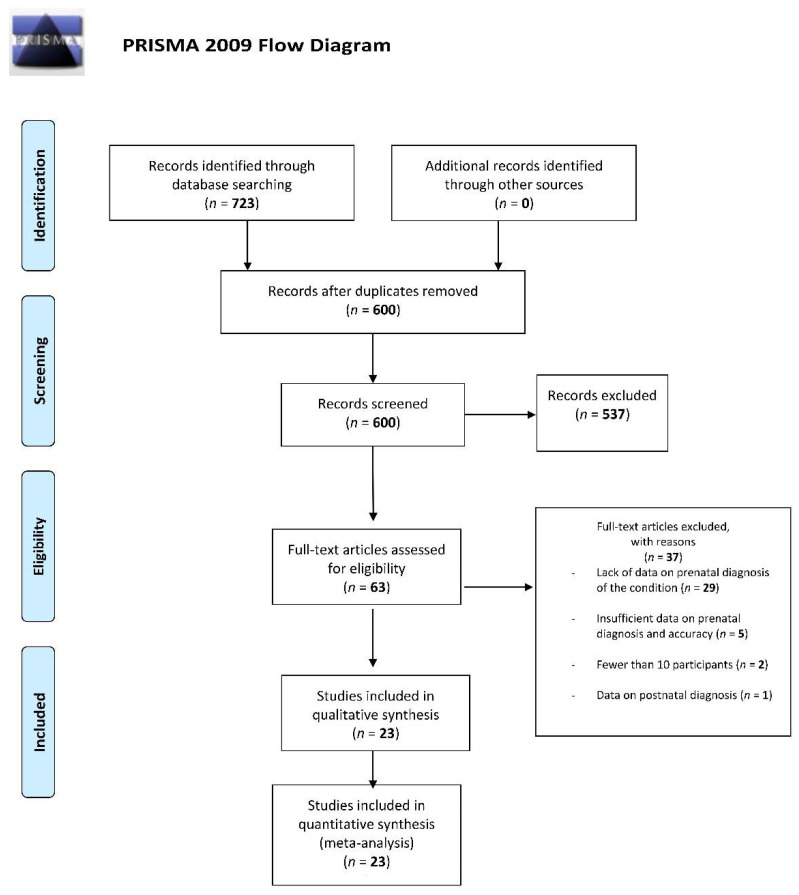
PRISMA flow diagram for studies selection.

The literature search retrieved 723 articles; upon checking for duplicates, 600 articles remained. Of the 600 articles, 539 were excluded based on title and abstract screening. A total of 62 articles were screened in full text and 37 were excluded mainly due to lack of data on prenatal diagnosis of the condition (*n* = 29), but also because of insufficient data on prenatal diagnosis and accuracy (*n* = 5), fewer than 10 participants (*n* = 2), and data on postnatal diagnosis (*n* = 1). Thus, at the final screening, 23 articles met the selection criteria and were included in this review. The screening process is reported in Figure 1.

### 3.2. Study Characteristics

The 23 selected studies included a total of 2318 patients who were prenatally diagnosed with clubfoot by US and/or MRI, some included karyotyping through amniocentesis and/or CVS. 11 of the studies included information about whether or not the diagnosis was confirmed after birth (accuracy) [1,10,11,12,13,14,16,22,23,25,27], the rest of the studies were used to obtain information about the main methodology used for prenatal diagnosis of clubfoot, but did not provide any detail about its accuracy; therefore, this item was not assessed. The minimum age was the first trimester of pregnancy (the week was not specified) [8,18,23,25,26] while the maximum reported age was 28 weeks of pregnancy [20]. 

The final studies selected by the reviewers included the following levels of evidence: 19 level III retrospective studies [1,2,7,9,10,12,13,14,15,16,17,18,21,22,25,26,27], 3 level II prospective comparative studies [8,11,20], 1 descriptive study of level III [19] (Table 1). 

### 3.3. Quality of Evidence

**Figure 2:**
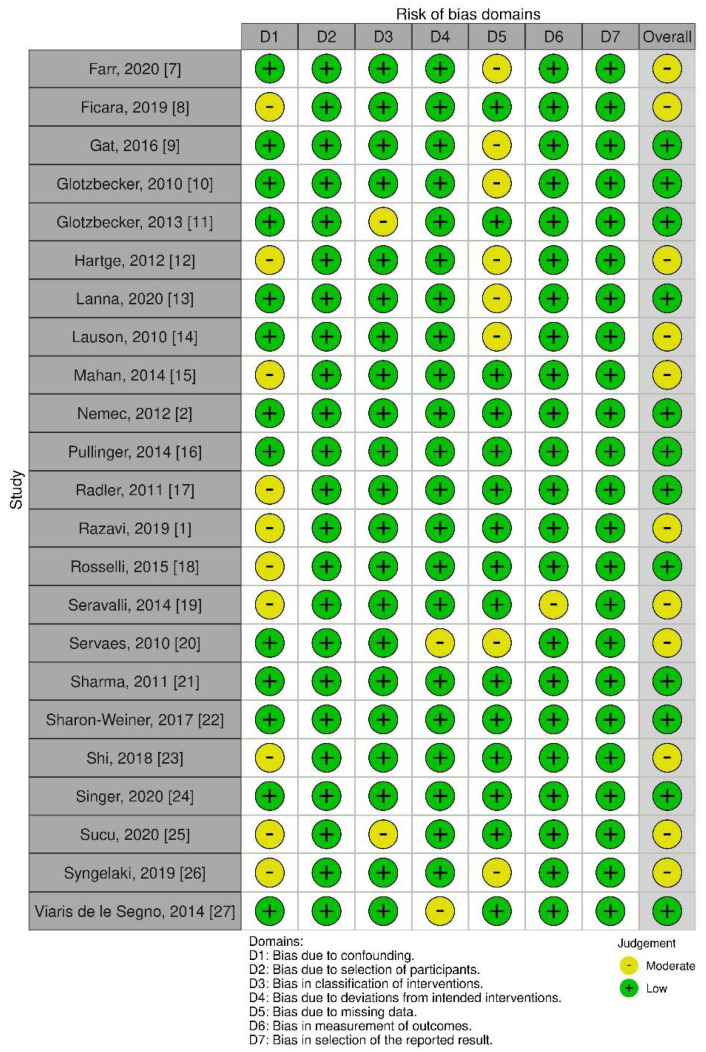
The risk of bias assessments for NRCTs studies with ROBINS-I Diagram.

Using the ROBINS-I tool 12 studies were scored as having a “low risk of bias” while 11 had a “moderate risk of bias” (Figure 2). The most common bias domains included “bias due to confounding” and “bias due to missing data”. The studies reviewed were similar in design and often lost follow-up or did not evaluate fully for possible confounding domains across variables. The RoB-2 tool was not used as Randomized Studies were not included.

### 3.4. Associated Pathologies

Six of the studies identified reported associated pathologies [9,14,21,24,25,27]. The following were mentioned in at least 1 of the studies included; Trisomies: 13, 18, 21, Neural Tube Defect, Skeletal Dysplasia, Cardiac anomalies, Sex Chromosome Abnormalities, Submicroscopic CNVs, Developmental Delay, Symptomatic Epilepsy, Thin corpus callosum, Visual inattentiveness, Peroneal Nerve Palsy, Low weight gain, Mild facial asymmetry, Delayed bone age, Cleft Palate, Finger Camptodactyly, Unusual facies, Cerebral Palsy, Rett Syndrome, Hypotonia, Coarse facial features, Torticollis, Hydramnios, Spina Bifida (Table 2). 

### 3.5. Diagnostic Procedure 

The diagnostic procedure involved US screening and MRI either in combination or individually. In addition, karyotyping (through amniocentesis and/or CVS) is also used in some cases to complete the diagnosis, not to screen directly for the condition. Diagnostic timing varied across the studies considered (Table 1).

### 3.6. Timing of Diagnosis

A total of 13 of the studies specified the timing at which the diagnosis was made [7,8,13,16,18,21,22,23,24,25,26,27]. Of the 13 studies that specified timing of diagnosis, 5 included diagnosis during trimester 1 [8,18,23,25,26], and this was the minimum age of diagnosis reported. Five studies stated that diagnosis occurred during trimester two or three [13,16,20,21,22]. The maximum age of diagnosis was reported by Servaes [20] as week 28. Of the studies that diagnosed patients in different trimesters Sucu [25], Syngelaki [26], and Rosselli [18] all had the fewest diagnosis during the first trimester, 10/138, 2/92, and 8/60, respectively, while Ficara [8] diagnosed 60 patients in trimesters 1 and 2 and only 1 in trimester 3 (Table 1).

### 3.7. Ultrasound

In all of the selected studies US was used as the primary diagnostic instrument. Thirteen of the studies [1,7,8,10,11,12,15,16,17,19,21,23,26] used the US exclusively, while in the rest of the studies [2,9,13,14,18,20,22,24,25,27] US was used in combination with other procedures such as MRI [2,9,20] or karyotyping [13,14,18,22,24,25,27] (Table 1). Based on the data available and on the studies in which this item was present, the accuracy of this test averaged 80.9%. 

### 3.8. Magnetic Resonance Imaging 

Of the 23 studies, 3 [2,9,20] used MRI in addition to the US to confirm the Clubfoot diagnosis and/or identify associated abnormalities; combined, they screened 71 patients. While Nemec [2] and Servaes [20] screened all patients with a US diagnosis using MRI, Gat [9] only screened 14 patients, those with an uncertain US diagnosis (Table 1). This showed that MRI was primarily used as an additional diagnostic tool to confirm US diagnoses, especially in case of uncertainty. Accuracy of diagnosis in these studies was not available. 

### 3.9. Karyotyping

In six studies [9,14,21,24,25,27] some or all patients underwent karyotyping through amniocentesis and/or CVS, to identify possible genetic abnormalities associated with the clubfoot diagnosis made through the US and/or MRI. In total 588 patients with a US prenatal diagnosis underwent karyotyping. Of the seven studies which performed this procedure only Lanna [13] and Sharon-Weiner [22] performed it on all patients diagnosed with Clubfoot (Table 1). Those studies that performed MRI did not perform amniocentesis. 

### 3.10. Meta-Analysis Results

According to Sterne et al. [29], meta-analyses should include research with a low or moderate risk of bias, therefore all publications should be included in the quantitative analysis. However, only 12 [1,2,10,11,12,13,14,16,22,23,25,27] of the 23 articles were examined since they contained data on the accuracy. Two groups were compared in terms of accuracy, the first “Genetic” [13,14,22,25,27] in which all or some patients underwent the karyotype, and the second “Imaging” [1,2,10,11,12,16,23] in which all patients underwent only US or MRI. 

**Figure 3:**
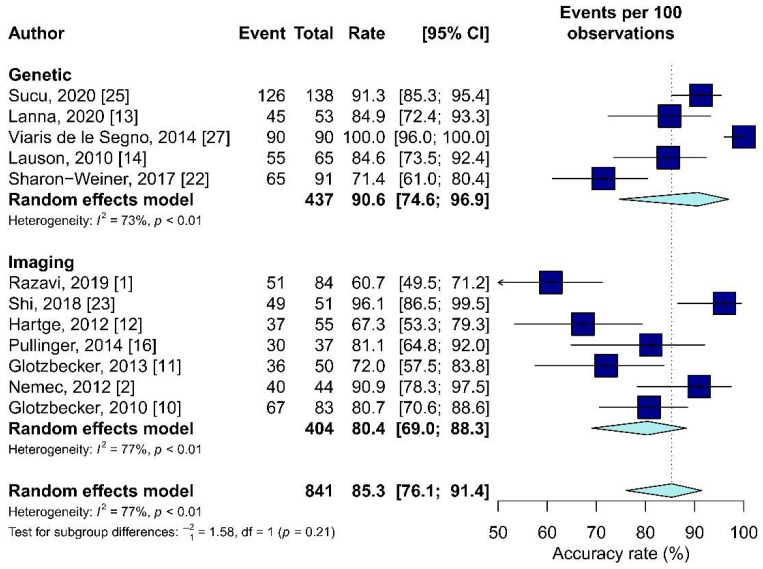
The forest plot of the accuracy rate in the “Genetic” and “Imaging” groups.

The average accuracy weighted was higher in the “Genetic” group (90.6% in the “Genetic” group and 80.4 in the “Imaging” group). However, no statistically significant differences were found between the two groups (*p* = 0.21, Figure 3).

## 4. Discussion

The present study reviewed the most recent literature to provide data about the most helpful tool for the prenatal diagnosis of clubfoot. The studies reviewed utilized US and MRI as methodologies for the prenatal diagnosis of clubfoot while, when performed, karyotyping was used to complete the diagnosis; however, US was favored. US was used in all 23 studies, compared to only three studies in which US was integrated with MRI and seven in which the practitioners proceeded with karyotyping after the diagnosis. The results reviewed seem to favor the US which can be identified as a better choice thanks to the growing level of accuracy, the safety of the non-invasive and non-radioactive procedure and the more advanced equipment. Instead, karyotyping could be helpful only to exclude other concomitant congenital diseases, while MRI does not provide further data to the diagnosis of congenital clubfoot. Moreover, no statistical differences in term of accuracy were reported between genetic and imaging diagnosis.

In the past, prenatal diagnosis of clubfoot raised several doubts due to a variable degree of inaccuracy. However, the sensitivity and specificity of different diagnostic methods have improved during the years [12,16,19]. Furthermore, a prenatal diagnosis helps the parents understand the pathology before birth, contributing to secure the parent-child bonding. Furthermore, it grants time to learn more about the condition and possible treatments, providing time to look for the best pediatric hospital [30,31]. Furthermore, it is possible to provide a multidisciplinary approach, including specialized orthopedics, a genetic counsellor, and other specialists if needed [15,32]. Therefore, providing evidence about the most useful diagnostic tool is mandatory to improve the advantages of a prenatal diagnosis.

Both Nemec et al. [2] and Servaes et al. [20] compared the US with MRI: according to Nemec et al., MRI screening has proved to be a valuable tool for clarification of doubtful US results or to add further findings after the US has been performed. However, the authors emphasized the advantages of MRI in complex CTEV, while in cases of isolated CTEV its utility is questionable. Servaes et al. present MRI as a tool capable of accurately detecting clubfoot; despite this, MRI should not be used as a preferential prenatal diagnostic procedure for clubfoot since its effects on the fetus are still unclear [6]. A comparison between the accuracy of ultrasound and MRI is not possible since the data for the latter technique is not specified in the reviewed studies, which adds to the unclear reliability of this methodology.

Despite being used for over four decades in various prenatal diagnoses and technological development, US diagnoses have some weaknesses. Results are strongly variable also depending on the age of pregnancy at the time of diagnosis [6]. Bogers et al. [33] even found that a diagnosis of clubfoot in the first trimester is not advisable due to the development of a transient clubfoot as a normal stage of development of the lower limb. These factors lower the accuracy of prenatal diagnosis of CTEV. This specific issue is discussed and partially solved by Glotzbecker [11] who suggest that a classification of the foot as “mild” “moderate” or “severe” may help to differentiate between a clubfoot, which will probably be confirmed at birth and one which may result in a false positive postnatally. 

The results of the present review are concordant with those of Faldini et al. [6], who also performed a review on the topic in 2016. However, the authors performed research from the inception between 1966 and 2015. Moreover, Faldini et al. [6] does not provide a detailed report for each study: data regarding sample size, accuracy, diagnosis timing, and associated pathologies are incomplete and described only for some of the included studies. Lastly, in the study by Faldini et al. [6], the Risk of Bias of the included studies was not reported, making it difficult to provide detailed results. 

### Limitations

Studies were conducted in different countries and hospitals, potentially causing a discrepancy in the type of equipment used. Finally, US diagnosis accuracy depends on the clinician’s skills, influencing the results. 

Widening the search before 2010 may have given a more comprehensive range of data. However, the present study updates a previous systematic review [6] which included studies from 1966 to 2015. Secondly, the US diagnosis of clubfoot has vastly improved in the last two decades thanks to the development of US technology and other diagnostic techniques [5]. A clear demonstration of this improvement is presented in a study published in 2007 that reported an improvement in US accuracy in prenatal diagnosis detection from 43% to 77% [34] in the last 18 years.

## 5. Conclusions

The results of the present systematic review and meta-analysis show that US has high level of accuracy, but comparable with MRI. However, US is not expansive and is a non-invasive procedure. Instead, karyotyping could be useful to exclude other diseases and MRI does not provide further data to the diagnosis of congenital clubfoot.

International guidelines for the prenatal diagnosis of clubfoot are needed and, in light of the current review, ultrasound appears to be the most suitable diagnostic methodology.

## Figures and Tables

**Table 1:** Primary author, year of publication, country, type of study, level of evidence (LOE), sample size, diagnosis, accuracy, sex, type of diagnosis and timing of the studies included.

Author, Year	Country	Type of Study, Level of Evidence	Sample Size	Diagnosis	Accuracy (%)	Sex (F/M)	Diagnosis				Timing
							Imaging		Genetic		
							MRI (*n*)	US (*n*)	A (*n*)	Other (*n*)	
Razavi, 2019 [1]	Germany	Retrospective Comparative Study, III	*	84	51/84 60.7%	29/55 (Sample size)	-	x	-	-	-
Nemec, 2012 [2]	Austria, USA	Retrospective Study, III	-	44	-	-	X ^$^	x	-	-	-
Farr, 2020 [7]	Austria	Retrospective Cohort Study, III	104	56	-	-	-	x	-	-	Mean: Week 20.5 ± 5.4
Ficara, 2019 [8]	UK	Prospective Comparative Study, II	52,400	61	-	-	-	x	-	-	T1 and T2 (60) T3 (1)
Gat, 2016 [9]	Israel	Retrospective Study, III	28	12	-	-	x (14)	x	-	-	-
Glotzbecker, 2010 [10]	USA	Retrospective Study, III	-	107 of which: 83 survived	67/83 80.7%	-	-	x	-	-	-
Glotzbecker, 2013 [11]	USA	Prospective Study, I	-	50	36/50 72.0%	-	-	x	-	-	-
Hartge, 2012 [12]	Germany	Retrospective Study, III	106, survived: 55	55	37/55 67.0%	-	-	x	-	-	-
Lanna, 2020 [13]	Italy	Retrospective Cohort Study, III	64	53	45/53 84.9%	-	-	x	X	-	T2 + T3 Follow-up or T3 only
Lauson, 2010 [14]	Canada	Retrospective Study, III	-	65	55/65 84.6%	-	-	x	x (41)	-	-
Mahan, 2014 [15]	USA	Retrospective Study, III	-	421		-	-	x	-	-	-
Pullinger, 2014 [16]	UK	Retrospective Comparative Study, III	-	74 of which: 37 found suitable for study	30/37 81.0%	-	-	x	-	-	Between Weeks 18 and 20
Radler, 2011 [17]	Austria, USA	Retrospective Study, III	-	92	-	-	-	x	-	-	-
Rosselli, 2015 [18]	Colombia	Descriptive, Retrospective Study, III	-	61	-	-	-	x	x (13)	-	T1 (8), T2 (38) T3 (14)
Seravalli, 2014 [19]	Italy	Descriptive Analysis	168	-	-	-	-	x	-	-	-
Servaes, 2010 [20]	USA	Prospective Study, I	13	-	-	-	X ^$^	x	-	-	Weeks 19–28
Sharma, 2011 [21]	UK	Retrospective Observational Study, III	174	-	-	-	-	x	-	-	Week 21
Sharon-Weiner, 2017 [22]	Israel	Retrospective Study, III	109 (51 bilateral; 58 unilateral) of which: 91 survived	91	65/91 71.4%	-	-	x	x	CVS	Weeks 14–16 or 21–24
Shi, 2018 [23]	China	Retrospective Study, III	4080	51	49/51 96.1%	-	-	x	-	-	Weeks 12–14
Singer, 2020 [24]	Israel	Retrospective Cohort Study, III	5750	269	-	-	-	x	x (Karyotyping and CMA, 229)	-	Mean: Week 22.6 ± 5.5
Sucu, 2020 [25]	Turkey	Retrospective Cohort Study, III	7680	138	126/138 91.3% (3FP in T1, 9FP in T2)	43/83 (Diagnosis)	-	x	x (83)	-	T1 (10) and T2 (128)
Syngelaki, 2019 [26]	UK	Retrospective Cohort Study, III	101,793	89	-	-	-	x	-	-	T1 (2), T2 (82), T3 (5)
Viaris de le Segno, 2014 [27]	France	Retrospective Study, III		90	90/90 100%	-	-	x	x (78)	-	Median: Week 23

F: Females; MRI: Magnetic Resonance Imaging; US: Ultrasound; A: Amniocentesis; * All Pregnancies scanned (ultrasound) at their institution from 2002 to 2014; ^$^: MRI confirmed ultrasound diagnosis; FP: False Positive; CVS: Chrionic Villus Sampling; T1, T2. T3: Trimester 1, 2, 3.

**Table 2:** Primary author, year of publication and associated pathologies of the studies included.

Author, Year	Associated Pathology
Lauson, 2010 [14]	Developmental Delay, Symptomatic Epilepsy, Thin corpus callosum, Visual inattentiveness, Peroneal Nerve Palsy, Low weight gain, Mild facial asymmetry, Delayed bone age, Cleft Palate, Finger Camptodactyly, Unusual facies, Cerebral Palsy, Rett Syndrome, Hypotonia, Coarse facial features, Torticollis.
Sharma, 2011 [21]	Brain, Heart, and Skeletal structural abnormalities, Hydramnios, Spina Bifida.
Singer, 2020 [24]	Chromosomal Aberrations, Submicroscopic CNVs, Trisomies: 18, 21, Sex Chromosome Abdnormalities.
Sucu, 2020 [25]	Trisomies: 13, 18, 21, Neural Tube Defect, Skeletal Dysplasia, Cardiac anomalies.
Viaris de le Segno, 2014 [27]	47, XY 1 18 (*n* = 4) 47, XX118 (*n* = 1) 46, XX der(8) t(8;11) (*n* = 1) Triploidy (*n* = 2) 46, XY inv(4) (*n* = 1) 47, XYY (*n* = 1)

CNV: Copy Number Variation.

## Data Availability

The data presented in this study are available on request from the corresponding author.
